# Clinical phenotype and outcomes of pneumococcal versus meningococcal purpura fulminans: a multicenter retrospective cohort study

**DOI:** 10.1186/s13054-021-03812-1

**Published:** 2021-11-11

**Authors:** Damien Contou, Nicolas de Prost, the HOPEFUL Study group, Laurent Argaud, François Barbier, Amélie Bazire, Gaëtan Béduneau, Frédéric Bellec, Pascal Beuret, Pascal Blanc, Cédric Bruel, Christian Brun-Buisson, Gwenhaël Colin, Delphine Colling, Alexandre Conia, Rémi Coudroy, Martin Cour, Damien Contou, Fabrice Daviaud, Vincent Das, Jean Dellamonica, Nadège Demars, Stephan Ehrmann, Arnaud Galbois, Elodie Gelisse, Julien Grouille, Laurent Guérin, Emmanuel Guérot, Samir Jaber, Caroline Jannière, Sébastien Jochmans, Mathieu Jozwiak, Pierre Kalfon, Antoine Kimmoun, Alexandre Lautrette, Jérémie Lemarié, Charlène Le Moal, Christophe Lenclud, Nicolas Lerolle, Olivier Leroy, Antoine Marchalot, Bruno Mégarbane, Armand Mekontso Dessap, Etienne de Montmollin, Frédéric Pène, Claire Pichereau, Gaëtan Plantefève, Sébastien Préau, Gabriel Preda, Nicolas de Prost, Jean-Pierre Quenot, Sylvie Ricome, Damien Roux, Bertrand Sauneuf, Matthieu Schmidt, Guillaume Schnell, Romain Sonneville, Jean-Marc Tadié, Yacine Tandjaoui, Martial Tchir, Nicolas Terzi, Xavier Valette, Lara Zafrani, Benjamin Zuber

**Affiliations:** 1 Service de Réanimation Polyvalente, Centre Hospitalier Victor Dupouy, 69, rue du Lieutenant-Colonel Prudhon, 95100 Argenteuil, France; 2 Service de Médecine Intensive Réanimation, Groupe de Recherche CARMAS, Centre Hospitalier Universitaire Henri Mondor, Assistance Publique-Hôpitaux de Paris, 51, avenue du Maréchal de Lattre de Tassigny, 94010 Créteil, France

**Keywords:** Purpura fulminans, *Neisseria meningitidis*, *Streptococcus pneumoniae*, Septic shock, Meningitis

Purpura fulminans (PF) is a rare cause of septic shock characterized by the association of a sudden and extensive purpuric rash together with an acute circulatory failure [1] leading to high rates of intensive care unit (ICU) mortality [1, 2] and long-term sequelae [3]. Clinical presentation of patients with PF differs from that of patients with meningitis since PF patients are commonly admitted to the ICU for hemodynamic impairment exposing them to early death from refractory circulatory failure, as opposed to patients with meningitis who are usually admitted to the ICU for neurological impairment. Among adult patients, *Neisseria meningitidis* and *Streptococcus pneumoniae* are the most commonly involved microorganisms accounting for more than 80% of PF [1] and meningitis [4]*.* While clinical features and outcomes widely differ between adult patients with pneumococcal and meningococcal meningitis [4], it remains unclear whether pneumococcal (pPF) and meningococcal (mPF) PF exhibit different clinical phenotypes and outcomes, although pPF was previously shown to predominantly occur in asplenic patients [5] and carries a higher risk of limb amputation [1]. We therefore compared the clinical, biological presentations and outcome of adult patients with pPF and mPF.

We performed an ancillary analysis of a 17-year multicenter retrospective study conducted in 55 centers in France, which included all consecutive patients (≥ 18 years) admitted to the ICU for an infectious PF (2000–2016) [1]. Patients with non-microbiologically documented PF or a bacterial documentation other than *Neisseria meningitidis* and *Streptococcus pneumoniae* were excluded.

During the study period, 195 patients with mPF and 67 with pPF were included. As compared to patients with mPF, those with pPF were older and had higher ICU severity scores. Chronic alcoholism and asplenia were more frequent in pPF, while the proportion of patients without previous comorbid conditions was lower. The time elapsed between disease onset and ICU admission was longer and purpura was less often noticed before ICU admission in pPF than in mPF. pPF patients also had lower platelet counts, higher serum urea and creatinine levels, and more frequent bacteremia. pPF patients needed more frequent invasive mechanical ventilation support, renal replacement therapy, plasma and platelets transfusions and had higher durations of invasive mechanical ventilation and vasopressor support. ICU mortality and rate of limb amputation were higher in patients with pPF (Table 1).

**Table 1:** Comparison between meningococcal (*n* = 195) and pneumococcal (*n* = 67) purpura fulminans

	Meningococcal purpura fulminans *n* = 195	Pneumococcal purpura fulminans *n* = 67	*p* value
*Patient’s characteristics and ICU scores*			
Male gender	97 (50)	37 (55)	0.527
Age, years	24 [19–45]	49 [38–60]	< 0.001
SAPS II	50 [35–66]	63 [58–72]	< 0.001
SOFA	11 [8–14]	14 [11–15]	< 0.001
*Main comorbidities*			
Chronic alcoholism	5 (2)	9 (13)	0.002
Diabetes mellitus	3 (2)	4 (6)	0.073
Asplenia or hyposplenia	3 (2)	34 (51)	< 0.001
Malignant hemopathy	1 (1)	2 (3)	0.162
Chronic respiratory disease	18 (23)	14 (28)	0.625
Immunocompromised status	5 (3)	4 (6)	0.241
No coexisting comorbid conditions	164 (84)	22 (33)	< 0.001
*Clinical features upon ICU admission*			
Days between disease onset and ICU admission, days	4 [4–5]	5 [4–6]	0.003
Headache	99 (51)	26 (39)	0.121
Myalgia	48 (25)	12 (18)	0.338
Digestive signs	124 (64)	41 (61)	0.839
Coma Glasgow score	15 [13–15]	15 [13–15]	0.751
Temperature, °C	38.5 [37–40]	38.5 [37–39]	0.802
Neck stiffness	52 (27)	6 (9)	0.004
Purpuric rash before ICU admission	168 (86)	38 (57)	< 0.001
β-Lactam antibiotic therapy before ICU admission	157 (81)	46 (69)	0.067
β-Lactam antibiotic therapy at ICU admission	195 (100)	67 (100)	–
*Biological data upon ICU admission*			
Leukocytes count, 10^3^ mm^−3^	10,700 [4000–20,800]	10,655 [2500–19,750]	0.717
Platelets count, 10^3^ mm^−3^	61,000 [28,500–100,000]	33,000 [19,000–49,500]	< 0.001
C-reactive protein, g/L	148 [90–247]	179 [141–289]	0.095
Procalcitonin, ng/mL	48 [14–100]	102 [55–164]	0.087
Troponin, mg/L	1 [0.10–12]	0.25 [0.13–11]	0.697
Creatine kinase, IU/L	300 [110–852]	812 [365–3460]	0.016
Serum urea, mmol/L	9 [7–11]	13 [11–15]	< 0.001
Serum creatinine, μmoL/L	190 [136–250]	240 [184–310]	< 0.001
Prothrombin time, %	33 [22–44]	29 [15–38]	0.227
Factor V, %	23 [10–49]	21 [9–29]	0.246
Arterial lactate, mmol/L	7.40 [5–11]	8 [6–11]	0.798
Fibrinogen, g/L	1.70 [0.6–3]	1.16 [0.5–2]	0.122
*Microbiological data at ICU admission*			
Bacteremia	99 (51)	56 (84	< 0.001
Lumbar puncture performed	125 (64)	29 (43)	0.004
Positive cerebro-spinal fluid culture	72/125 (58)	11/29 (38)	0.080
*Outcome in the ICU*			
Lowest LVEF, %	33 [20–45]	30 [25–50]	0.870
Inotropic agent	91 (64)	35 (61)	0.894
Platelets transfusion	57 (29)	46 (69)	< 0.001
Plasma transfusion	67 (34)	44 (66)	< 0.001
Steroids for septic shock or meningitis	116 (60)	45 (67)	0.333
Activated protein C	33 (17)	9 (13)	0.632
Invasive mechanical ventilation	152 (78)	65 (97)	0.001
Duration of tracheal intubation, days	4 [2–9]	10 [3–28]	< 0.001
Duration of vasopressors, days	3 [2–5]	5 [3–8]	< 0.001
Renal replacement therapy	69 (36)	45 (67)	< 0.001
Veno-arterial ECMO	7 (4)	6 (9)	0.104
Limb amputation	19 (10)	21 (31)	< 0.001
Limb amputation among ICU survivors	18/125 (14)	19/32 (59)	< 0.001
Death in ICU	70 (36)	35 (52)	0.027
Duration of ICU stay, days	5 [2–11]	14 [3–35]	< 0.001
Duration of hospital stay, days	12 [2–23]	23 [3–78]	0.003

Continuous variables are reported as median [Interquartile range] and compared between groups using the Student *t*-test. Categorical variables are reported as numbers (percentages) and compared using *χ*^2^ test. A *p* value < 0.05 was considered significant

*ICU* intensive care unit; *IMV* Invasive Mechanical Ventilation, *ECMO* Extracorporeal membrane oxygenation, *LVEF* Left ventricular ejection fraction, *SAPSII* Simplified Acute Physiology Score, *SOFA* Sequential Organ Failure Assessment

The Kaplan–Meier survival analysis did not show significant difference between pPF and mPF patients (*p* = 0.80 by the log-rank test, Fig. 1).

**Fig. 1:**
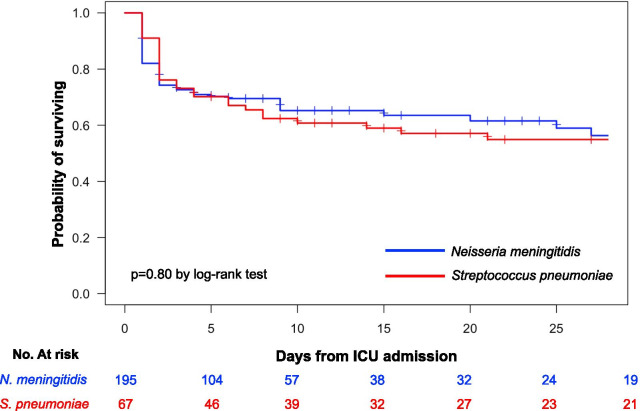
Kaplan–Meier survival estimates during the 30 days following ICU admission of patients with pneumococcal (red curve) and meningococcal (blue curve) purpura fulminans

By multiple logistic regression adjusting on age, SOFA score, administration of β-lactam antibiotic therapy before ICU admission, platelet counts and arterial lactate levels, pPF was not associated with ICU mortality (adjusted Odds Ratio = 1.15 95% CI 0.45–2.89, *p* = 0.77).

As already reported in adults patients with bacterial meningitis [4], this study confirms that significant differences exist between mPF and pPF, regarding both the clinical presentation at ICU admission and outcomes. Patients with pPF showed a different clinical phenotype, with less frequent purpura possibly leading to less frequent antibiotic treatment, more comorbidities with a more severe presentation at ICU admission, resulting in a higher rate of organ failures during ICU stay. Whether this more severe presentation should be ascribed to the level of virulence of the causative pathogen or to host-related characteristics is unsettled.

Our study has several limitations including its retrospective design and its long recruitment period with a high number of centers implying ICU procedures being inevitably heterogeneous. Nevertheless, the clinical presentation as well as the course in the ICU of patients with PF seem to differ according to the causative bacterium. This clinical observation should encourage researchers to better study the pathophysiology of pPF in order to develop targeted innovative therapies as being done for mPF [6].

## Data Availability

The dataset used and analyzed for the current study is available from the corresponding author on reasonable request.

## Abbreviations

ICU: Intensive care unit
mPF: Meningococcal purpura fulminans
pPF: Pneumococcal purpura fulminans
SOFA: Sequential Organ Failure Assessment
